# Hepatectomy for liver metastases from gastric cancer: a systematic review

**DOI:** 10.1186/s12893-017-0215-0

**Published:** 2017-02-13

**Authors:** Ying-Yang Liao, Ning-Fu Peng, Di Long, Peng-Cheng Yu, Sen Zhang, Jian-Hong Zhong, Le-Qun Li

**Affiliations:** 1 Nutrition Department, Affiliated Tumor Hospital of Guangxi Medical University, Nanning, China; 2 Hepatobiliary Surgery Department, Affiliated Tumor Hospital of Guangxi Medical University, Nanning, 530021 Guangxi Province China; 3 Colorectal Anal Surgery Department, The First Affiliated Hospital of Guangxi Medical University, Nanning, 530021 China

**Keywords:** Gastric cancer, Hepatectomy, Liver metastases, Systematic review

## Abstract

**Background:**

Official guidelines recommend palliative treatments for patients with liver metastases from gastric cancer. However, many case series reported that hepatectomy for such cases is safe and effective. This systematic review compares the overall survival between hepatectomy and palliative therapy in patients with liver metastases from gastric cancer.

**Methods:**

Two independent reviewers performed a systematic search of literature in EMBASE and PubMed, updated until 26 October 2016. The Newcastle-Ottawa score for cohort studies was used for quality assessment of included studies.

**Results:**

A total of eight cohort studies involving 196 patients in the hepatectomy arm and 481 in the palliative arm were included. Median overall survival of patients in the two arms was 23.7 (range, 13.0 to 48.0) and 7.6 (range, 5.5 to 15.2), respectively. Median rates of overall survival of the two arms were 69, 40, 33 and 27, 8, 4% at 1, 2, and 3 years, respectively. Comparing with palliative therapy, hepatectomy was associated with significantly lower mortality at 1 year (odds ratio 0.17, *P* < 0.001) and 2 years (odds ratio 0.15, *P* < 0.001). Among the patients who underwent hepatectomy, Asian cohorts showed higher median rates of overall survival than Western cohorts at 1 year (76 vs. 60%), 2 years (47 vs. 30%) and 3 years (39 vs. 23%).

**Conclusions:**

Hepatectomy in the management of liver metastases from gastric cancer can be considered effective. In the elective setting, hepatectomy provides a potential alternative to palliative therapy.

## Background

As the sixth highest incidence and the second leading cause of cancer deaths worldwide, gastric cancer is the most common form of cancer, with more than 951,000 new cases worldwide diagnosed in 2012 [1]. Due to late onset and nonspecific symptoms, the majority of gastric cancer cases present in advanced stage, with less than 30% of patients eligible for curative resection [2]. In recent decade, treatment of gastric cancer has been significantly improved, and the 5-year overall survival of patients with T1 tumors is up to 95% [3]. However, the prognoses of patients represented by peritoneal or liver metastases is extremely poor, with a 3-year overall survival lower than 10% after chemotherapy [4, 5].

Gastrectomy is more used in Eastern centers than Western centers. And Eastern patients’ prognoses after gastrectomy are better than those in Western [6]. According to the guideline of the Japanese Classification of Gastric Carcinoma [7], liver metastases from gastric cancer is categorized as stage IV disease. This guideline [7] and the National Comprehensive Cancer Network guideline [8, 9] do not recommend surgery for stage IV gastric cancer, which lead to most patients with liver metastases of gastric cancer receive palliative treatment (such as chemotherapy). Though the necessity of hepatectomy for liver metastases of gastric cancer is still controversial, the Guidelines Committee of the Japan Gastric Cancer Association reconsidered the treatment of potentially resectable M1 disease [10].

Recently, many case-control or case series studies of liver metastases from gastric cancer have been reported. However, most of these studies are presented from a single center, have a small sample size, and/or include old cases from the 1960s and 1980s [11–20]. Therefore, it is important to thoroughly analyze the significance of hepatectomy for liver metastases from gastric cancer, and compare the efficacy of hepatectomy with palliative therapy for such patients in recent two decades (1990–2016) with a systematic review.

## Methods

Two independent authors performed a systematic review (Y.-Y.L, D.L) according to the Preferred Reporting Items for Systematic Reviews and Meta-Analyses (PRISMA) statement [21]. A study protocol was followed which defined the study objectives, eligibility criteria, outcome measures, search strategy and methodology of analysis. The quality of included studies was assessed using the Newcastle-Ottawa score for cohort studies [22]. This tool was chosen because of the unavailability of randomized controlled trials and large heterogeneity between studies.

### Search strategy

A systematic search of literature, updated until 26 October 2016, was performed by two independent reviewers (Y.-Y.L, D.L). The EMBASE and PubMed databases were searched using MeSH and free text words. MeSH and free text words concerning gastric cancer and metastasis were used. No language or time period restrictions were applied. Titles and abstracts retrieved from the search were screened for relevance and selected studies. Disagreements during the search and selection process were resolved by discussion and, if needed, a third reviewer (N.-F.P) was consulted to reach consensus. Reference lists of all included articles were screened for additional eligible papers.

The following search strategy was used in PubMed (Medline):

((“cancer” [Mesh] AND “gastric Neoplasms” [Mesh]) OR “neoplasm, stomach” [Mesh] OR stomach neoplasm*[tw] OR gastric neoplasm*[tw] OR cancer of stomach*[tw] OR stomach cancer*[tw] OR gastric cancer*[tw]) AND (“Metastases, Neoplasm” [Mesh] OR metastasis*[tw] OR metastases*[tw]) AND (surgery*[tw] OR resection*[tw] OR hepatectomy*[tw]) AND (hepatic*[tw] OR liver*[tw]).

### Eligibility criteria and data extraction

Criteria for final study inclusion were: (1) study population formed by patients with liver metastases from gastric cancer in the absence of peritoneal metastasis or extractable from studies in which hepatectomy was performed; (2) sufficient description of the study population; (3) description of patient survival rates for at least 1 year after hepatectomy; (4) the study had a randomized control, cohort, or case-control design. In order to reduce the bias of diagnosis, original treatments, neoadjuvant and adjvuant treatments, only cases after 1990 may be included. In cases in which a study was followed by a more complete study or studies that included the original data set, the most recent and complete report was chosen. Such linked studies were identified on the grounds of authorship, institutions, design, length of follow-up, and study populations. If additional data or results were needed, the corresponding author of each report was contacted by e-mail.

Data were extracted by two authors (Y.-Y.L, D.L) using standardized forms. The following data were collected: author details, country, recruitment period, study design, median follow-up, sample size, gender, positive and negative findings, and methodological quality. A third author (N.-F.P) checked the extracted data against the original studies. Survival data were taken directly from tables or the text whenever possible; if such data were presented only in graphs, they were extracted by manual interpolation. *P* values associated with inter-group differences in mortality were extracted directly from survival curves, text, or tables wherever possible.

### Statistical analysis

Meta-analysis was performed on an intention-to-treat basis. To assess attrition bias, we calculated mortality using a ‘worst-case’ approach in which patients with missing data were counted as treatment failures (death). For patients with missing data, we ’carried forward’ data from the most recent measurement.

Outcomes are displayed as they were reported in the original article. All statistical tests for this meta-analysis were performed using Stata 11.0 softwares. Due to the high likelihood of mortality, odds ratio (ORs) with corresponding 95% confidence intervals (95% CIs) were calculated for dichotomous outcomes using the Mantel-Haenszel method. Point estimates of RR were considered statistically significant when *P* < 0.05. Statistical heterogeneity was explored by inconsistency (*I* ^2^) statistics; in particular, *I* ^2^ values of <25% was interpreted as low heterogeneity, between 25 and 50% as medium, between 50 and 75% as substantial and above 75% as considerable [23].

## Results

**Fig. 1:**
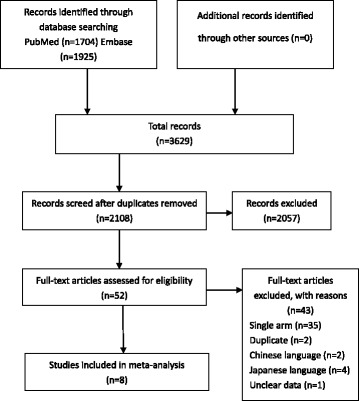
Flow chart of study selection

A total of 3629 records were identified. Following screening for duplications, 2108 articles remained. After screening by title and abstract, a further 2057 records were excluded. Of the remaining 52 articles which need full-text assessment, 35 were studies with single arm investigating the role of hepatectomy for liver metastases from gastric cancer [11–20, 24–48]. In the end, only 8 studies compared the efficacy of hepatectomy to that of other palliative treatments met the eligibility criteria and were included in a quantitative synthesis [49–56]. Details are listed in Fig. 1.

### Baseline characteristics

Five of the studies based on Eastern population [49, 50, 52, 53, 56] while the other three based on Western population [51, 54, 55]. These eight studies included 196 patients in the hepatectomy arm and 481 in the palliative arm. Most patients in the hepatectomy arm (70%) underwent minor hepatectomy. No study described complications during or after hepatectomy. Patient demographics and characteristics of liver metastases from gastric cancer are summarized in Table 1. The median follow-up was 13.2 months.

**Table 1:** Main characteristics of the retrieved literature regarding hepatectomy for hepatic metastases from gastric cancer

Study	Country	Recruitmentperiod	Sample size (hepatectomy/palliative therapy)	Hepatectomy arm					
				Median follow-up, mo	Median age, yr	Male/Female	Single/Multinodular	Uni/Bilobar	Synchronous/Metachronous
Chen 2013	China	2007–2012	20/94	10	57	12/8	8/12	11/9	20/0
Cheon 2008	Korea	1995–2005	41/17	16	60	34/7	28/13	NR	30/11
Dittmar 2012	Germany	1995–2009	15/83	11	57	12/3	8/7	12/3	9/6
Makino 2010	Japan	1992–2007	16/47	NR	NA	13/3	9/7	11/5	9/7
Miki 2012	Japan	1995–2009	25/25	NR	72	23/2	18/7	20/5	16/9
Tiberio 2009	Italy	1990–2004	11/62	19	NR	NR	NR	NR	0/11
Tiberio 2015	Italy	1997–2011	53/142	NR	NR	NR	NR	NR	53/0
Ueda 2009	Japan	1991–2005	15/11	NR	NR	NR	NR	NR	NR

*NR* not reported

### Efficacy

Median overall survival of patients in the hepatectomy arm and those in the palliative arm was 23.7 (range, 13.0 to 48.0) and 7.6 (range, 5.5 to 15.2), respectively. Median rates of overall survival of the two arms were 69, 40, 33 and 27, 8, 4% at 1, 2, and 3 years, respectively (Table 2).

**Table 2:** Overall survival of patients with hepatic metastases from gastric cancer after hepatectomy or palliative treatments

Study	Hepatectomy arm				Palliative arm				*P*
	Median survival (moths)	1-year	2-year	3-year	Median survival (moths)	1-year	2-year	3-year	
Chen 2013	22.3	70	30	21	5.5	15	0	0	<0.001
Cheon 2008	18.3	75	38	32	8.1	29	0	0	<0.001
Dittmar 2012	48.0	79	64	55	9.0	42	18	9	<0.001
Makino 2010	38.2	87	68	56	15.2	53	12	4	<0.001
Miki 2012	33.4	74	55	43	8.7	40	18	18	0.045
Tiberio 2009	23.0	80	30	20	6.9	28	6	3	<0.001
Tiberio 2015	13.0	50	21	14	5.5	13	7	2	<0.001
Ueda 2009	-	80	60	60	10.0	36	0	0	<0.001

**Fig. 2:**
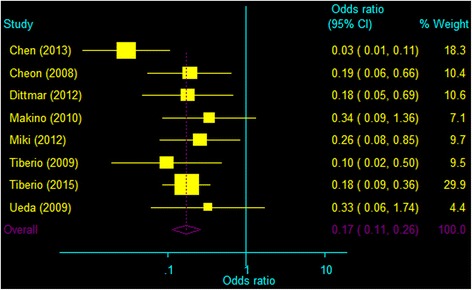
Meta-analysis of mortality at 1 year after hepatectomy or palliative therapies in patients treated for liver metastases from gastric cancer

**Fig. 3:**
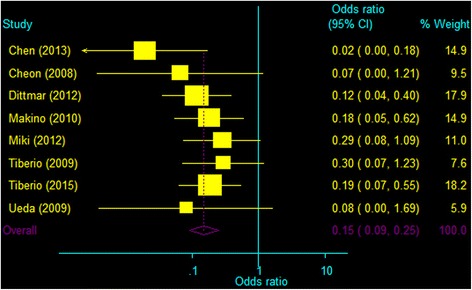
Meta-analysis of mortality at 2 years after hepatectomy or palliative therapies in patients treated for liver metastases from gastric cancer

Comparing with palliative therapy, hepatectomy was associated with significantly lower mortality at 1 year (OR 0.17, 95% CI 0.11–0.26, *P* < 0.001; Fig. 2) and 2 years (OR 0.15, 95% CI 0.09–0.25, *P* < 0.001; Fig. 3).

Among the patients who underwent hepatectomy, comparison of studies performed in Asian countries (5 studies, 117 patients) and Western countries (3 studies, 79 patients) indicated that Asian cohorts showed higher median rates of overall survival at 1 year (76 vs. 60%), 2 years (47 vs. 30%) and 3 years (39 vs. 23%).

### Quality assessment

Details on individual quality of studies are displayed in Table 3. Among the 8 cohort studies, only one had eight stars [50], one had seven stars [52], and the remaining 6 studies had six stars [49, 51, 53–56].

**Table 3:** Newcastle-Ottawa score quality assessment scale for cohort studies

Study	Selection				Comparability		Outcome			Total star
	1	2	3	4	1a	1b	1	2	3	
Chen 2013	a*	a*	a*	a*	a*	b*	d	b	d	6
Cheon 2008	a*	a*	a*	a*	a*	b*	d	a*	a*	8
Dittmar 2012	a*	a*	a*	a*	-	-	d	a*	a*	6
Makino 2010	a*	a*	a*	a*	a*	b*	d	a*	d	7
Miki 2012	a*	a*	a*	a*	a*	-	d	a*	d	6
Tiberio 2009	a*	a*	a*	a*	-	-	d	a*	a*	6
Tiberio 2015	a*	a*	a*	a*	-	-	d	a*	a*	6
Ueda 2009	a*	a*	a*	a*	-	-	d	a*	a*	6

-, no description or comparability

## Discussion

Among all included studies in this systematic review, a total of 196 patients were treated with hepatectomy for liver metastases from gastric cancer. Hepatectomy was associated significantly better overall survival than palliative therapy for such patients. Our results were consistent with previous reviews [57–60].

The liver is a common site of distant metastasis from gastrointestinal tract cancer, including gastric cancer. Hepatectomy is now widely accepted as a potentially curative treatment for hepatocellular carcinoma [61, 62] and colorectal liver metastases [63], with reported 5-year overall survival of 40–50% [61–63]. Moreover, indications of hepatectomy for hepatocellular carcinoma and colorectal liver metastases have been expanded by progress in surgical procedures, perioperative care and/or chemotherapy. Liver metastases from gastric cancer not only show more aggressive oncological behavior and heterogeneous characteristics, but also are with frequently other metastatic extrahepatic lesions, such as peritoneal seeding or extensive lymph node metastases, leading to a dismal prognosis and debatable benefits of hepatectomy. Therefore, chemotherapy is regarded as the first-choice treatment in most guidelines. However, a lot of case reports and case series supported the role of hepatectomy for liver metastases from gastric cancer [11–20, 24–48], which lead to Japanese guideline reconsidered the role of hepatecotmy for such patients [10].

Previous systematic reviews [57–60] were mainly based on some part of the case series [11–20, 24–48]. Many of the quoted and included studies of these systematic reviews are of small studies stretching back in some cases to the 1960’s. This leads to many concerns over the diagnosis, original treatments, neoadjuvant and adjuvant treatments in such a big time span. Assessment of metastatic disease in the pre multilevel computer tomography and positron emission tomography era is also doubtful when trying to compare outcomes. In addition, those systematic reviews are not comparing like with like. Those undergoing hepatectomy are a highly selected group with resectable liver disease in the absence of other disease. It is not reasonable to compare this with all comers with widespread metastatic disease. It would be more reasonable to cone in on those studies where comparative outcomes were considered. Therefore, we only focus on cohort studies which comparing hepatectomy and palliative therapy and patients who were included after 1990. Such results may be more reliable than previous.

Our systematic review revealed that median overall survival after hepatectomy was 23.7 (range, 13.0 to 48.0). Median overall survival was ranges from 24 to 40.8 months after hepatectomy among other large scale case series (>60 cases) [40, 46–48]. Hepatectomy for liver metastases from gastric cancer is not performed frequently at present. It is notable that none of the patients who underwent hepatectomy in this systematic review or these case series [40, 46–48] was with peritoneal metastasis. Careful patient selection is likely to be important for ensuring good prognosis after hepatectomy. The number of hepatic tumors (≥3), tumor diameter (≥5), and serosal invasion of the primary tumor were identified as prognostic factors associated with a poor overall survival [19, 37, 40, 46]. Moreover, patients with more risk factors have much poor survival rates at 3 or 5 years after hepatectomy [46]. Therefore, palliative therapy should be considered when any of these factors is recognized at diagnosis. Other indications for hepatectomy included adequate physical condition, preserved liver function, and feasibility of complete tumor resection. In addition, indications for hepatectomy may also need to consider response rate to neoadjuvant chemotherapy in those patients who receive it, since prognosis of non-responders is generally worse than that of responders [64, 65].

The role of neoadjuvant or adjuvant therapy for liver metastases from gastric cancer is still controversial. Of the eight included studies, 16.4% patients received neoadjuvant therapy, while 55.2% received adjuvant therapy. If all case series [11–20, 24–48] and these eight cohort studies [49–56] were included into analysis, the percentage of patients who received neoadjuvant therapy for the primary gastric cancer was 11.3%, and the percentage of patients receiving adjuvant therapy was 55.1%. For patients with gastric cancer with extensive lymph node metastasis, Tsuburaya and coworkers found neoadjuvant chemotherapy with 4-weekly S-1 and cisplatin followed by D2 gastrectomy is safe and effective for some patients [66]. Further trials should be performed to investigate the role of neoadjuvant therapy as a biological trial.

We found Asian cohorts showed higher median rates of overall survival than Western cohorts. Western cohorts usually show more advanced stage of disease than Asian cohorts [19, 24, 28, 41, 43, 50, 51, 54, 55]. Such presentation may lead to more Western cases receive chemotherapy while more Asian cases receive curative resection. Even so, there needs to be consideration of the role of chemotherapy versus surgery in the chemotherapy naïve patients. In this systematic review, hepatectomy associated with a median overall survival of 23.7 months. However, median overall survival was only 11.3 to 13.8 months after combination therapy with or without trastuzumb for advanced gastric cancer [4, 67]. Therefore, our results suggest that hepatectomy is associated with substantially longer median overall survival than combination chemotherapy with or without targeted therapy. In selected patients, hepatectomy may be preferable to chemotherapy.

A limitation of our study is the low availability of high level evidence studies in literature. Since all studies were retrospective cohort studies, a potential bias is that the positive effects of hepatectomy are overestimated, as cases of unsuccessful resection are less likely to be reported or published. Moreover, the types and frequency of postoperative complications remain unclear because all of the studies failed to report such data. Most patients underwent minor rather than major hepatectomy. Heterogeneity in surgical technique and skill may also affect patient prognosis.

## Conclusions

This systematic review provides comprehensive evidence that hepatectomy is associated with longer median overall survival than palliative treatments for selected patients with liver metastases from gastric cancer. If our findings can be verified and extended in a completion of a randomized controlled trial on hepatectomy versus chemotherapy or a high-quality prospective study with long-term follow-up, they make a strong argument for changing current clinical practices and official guidelines to bring them into line with the evidence base.

## Abbreviations

CI: Confidence interval
OR: Odds ratio
